# Knowledge on cervical cancer and perceived barriers to the uptake of HPV vaccination among health professionals

**DOI:** 10.1186/s12905-021-01205-8

**Published:** 2021-02-12

**Authors:** Poongodi Chellapandian, Sindhura Myneni, Divya Ravikumar, Padmavathy Padmanaban, Kavin Mozhi James, Vahitha Mala Kunasekaran, Rejili Grace Joy Manickaraj, Christina Puthota Arokiasamy, Poonguzhali Sivagananam, Pandian Balu, Udayakumari Meesala Chelladurai, Vishnu Priya Veeraraghavan, Gayathri Baluswamy, Radhika Nalinakumari Sreekandan, Devakumar Kamaraj, Sumetha Suga Deiva Suga, Malathi Kullappan, Jenifer Mallavarapu Ambrose, Sai Ravi Teja Kamineni, Krishna Mohan Surapaneni

**Affiliations:** 1 Department of Obstetrics and Gynaecological Nursing, Panimalar College of Nursing, Varadharajapuram, Poonamallee, Chennai, Tamil Nadu 600 123 India; 2 Department of Obstetrics and Gynaecology, Panimalar Medical College Hospital and Research Institute, Varadharajapuram, Poonamallee, Chennai, Tamil Nadu 600 123 India; 3 Department of Medical Surgical Nursing, Panimalar College of Nursing, Varadharajapuram, Poonamallee, Chennai, Tamil Nadu 600 123 India; 4 Department of Community Health Nursing, Panimalar College of Nursing, Varadharajapuram, Poonamallee, Chennai, Tamil Nadu 600 123 India; 5 Department of Mental Health Nursing, Panimalar College of Nursing, Varadharajapuram, Poonamallee, Chennai, Tamil Nadu 600 123 India; 6 Medical Records Department (MRD), Panimalar Medical College Hospital and Research Institute, Varadharajapuram, Poonamallee, Chennai, Tamil Nadu 600 123 India; 7 Department of Biochemistry, Saveetha Dental College and Hospital, Saveetha Institute of Medical and Technical Sciences (SIMATS), Saveetha University, Velappanchavadi, Chennai, Tamil Nadu 600 077 India; 8 Departments of Clinical Skills and Simulation, Panimalar Medical College Hospital and Research Institute, Varadharajapuram, Poonamallee, Chennai, Tamil Nadu 600 123 India; 9 Department of Pharmacology and Division of Clinical Research - Department of Research, Panimalar Medical College Hospital and Research Institute, Varadharajapuram, Poonamallee, Chennai, Tamil Nadu 600 123 India; 10 Department of Microbiology, Panimalar Medical College Hospital and Research Institute, Varadharajapuram, Poonamallee, Chennai, Tamil Nadu 600 123 India; 11 Department of Research, Panimalar Medical College Hospital and Research Institute, Varadharajapuram, Poonamallee, Chennai, Tamil Nadu 600 123 India; 12 Department of Tuberculosis and Respiratory Diseases, Panimalar Medical College Hospital and Research Institute, Varadharajapuram, Poonamallee, Chennai, Tamil Nadu 600 123 India; 13 Departments of Biochemistry, Clinical Skills and Simulation and Research, Panimalar Medical College Hospital and Research Institute, Varadharajapuram, Poonamallee, Chennai, Tamil Nadu 600 123 India

**Keywords:** Cervical cancer, Prevention, Screening and human papillary tumour virus vaccination

## Abstract

**Background:**

Despite the fact that cervical cancer is preventable and curable in the early stages, it still remains to be a major public health problem in India. This study was conducted to assess the knowledge and awareness regarding the Human Papilloma Virus (HPV) vaccination among health care professionals working in a tertiary care hospital in urban India.

**Methods:**

To this aim, we conducted a cross-sectional study among 318 health care professionals working in tertiary hospitals across Chennai, Tamil Nadu, India. Our research group designed a structured questionnaire with 31 items to assess the knowledge and attitudes on cervical cancer, its prevention, and HPV vaccination.

**Results:**

Among the 318 respondents, 90.6% were aware of cervical cancer, 83.3% were aware that PAP (Papanicolaou) smear test detects cervical cancer, and 86.2% of the respondents knew that HPV causes cervical cancer. 29.2% of the eligible respondents underwent the screening against cervical cancer, and 19.8% of the study participants were vaccinated for HPV. Only 34.9% know that the HPV vaccine could be given to boys. The most common reason for not being vaccinated against HPV was the lack of awareness. In our study, 77.2% of the respondents were willing to be vaccinated and recommend HPV vaccination to their family members.

**Conclusion:**

From this study, it was evident that there is a lack of awareness about HPV vaccination and its importance in preventing cervical cancer among healthcare professionals. Our finding clearly establishes the need to devise intervention programs to promote vaccination against HPV and periodical screening for cervical cancer among healthcare professionals.

## Background

Cervical cancer is the fourth most commonly occurring cancer among women worldwide [1]. India accounts for one-fourth of the global burden of cervical cancer [2]. Unlike the other developed countries, cervical cancer remains a major public health problem and accounts for 17% of cancer deaths among women during their reproductive age group in India [1].

Human papillomavirus (HPV) infection causes cervical cancer, particularly HPV 16 and 18 strains account for 75% of these cases [3]. The risk factors for acquiring HPV infection include having multiple sexual partners, early age of sexual intercourse, tobacco consumption, prolonged use of oral contraceptive pills, increased parity, and early age of giving birth [4]. Most of the Genital HPV infections are asymptomatic but previous research evidence shows that nearly all cervical cancer cases are caused by high-risk HPV types [5]. Screening with Pap test or VIA(Visual Inspection with Acetic Acid) or effective HPV-DNA detection procedures can be employed to easily detect the precursors of cervical cancer at an early stage and treat them efficiently. Unlike cancers occurring in various sites of the body, cervical cancer, and its precursor lesions could be detected early, and is curable in the early stages of the disease. Screening women for cervical cancer is crucial as most of them do not often experience symptoms until the disease has advanced [5]. The prevention and control of cervical cancer depend on awareness about the disease, screening procedures, and preventive measures [4]. Despite being included under the non-communicable diseases control program by the Ministry of Health and Family Welfare (MoHFW), Government of India, there is still no organized or high opportunistic screening approaches implemented for cervical cancer in India.2

Though screening modalities have been in place for more than fifty years, the burden of the disease has not yet reduced as expected in India. For this reason, reinforcement with another preventive measure through vaccination has now been advocated. Bivalent and quadrivalent HPV vaccines are licensed for use in India [6]. The recommended age for vaccination is between 9 and 12 years. Catch-up vaccination is permitted up to the age of 26 years [7]. The quadrivalent vaccine is currently licensed and tested for usein males as well [8]. The vaccine is not recommended for use in pregnant women [7, 9]. Lactating women can also receive the vaccine. Vaccination is not a replacement to screening for cervical cancer. It is an added effort towards making the nation free of cervical cancer.

While the HPV vaccine has been in use for more than a decade in India, it is still not provided as an essential vaccine under the National Immunisation Programme by the Govt of India. Lack of awareness regarding its dosage, schedule, and cost exists even in a highly educated population in the health care sector. Health care professionals need to have complete and updated knowledge regarding the HPV vaccine for their own benefit and thus to promote the same among their patients. This study was conducted to assess the current awareness about cervical cancer and HPV vaccination among the health care professionals working in a tertiary care hospital and to overcome barriers associated with the uptake of the vaccine.

## Methods

### Study design, settings and participants

A Cross-sectional study was conducted at Panimalar Medical College Hospital & Research Institute, Chennai, India. A random sample of 318 health care professionals working in tertiary hospitals in Chennai, Tamil Nadu, India, was chosen for this study. Those participants who were above 18 years of age and willing to give their written consent to participate in this study have been included. We obtained written informed consent from all the participants. The study protocol was approved by the Institutional Review Board (IRB) of the Panimalar Medical College Hospital & Research Institute, Chennai (Panimalar Medical College Hospital & Research Institute IRB #1/2020/005) and conformed to the requirements of the Declaration of Helsinki (as revised in Seoul 2008).

### Variables and measures

The questionnaire/survey instrument was developed by our research team consists of thirty one questions/statements on demographic characteristics and information pertaining to the cervical cancer preventive methods. The socio-demographic variables embrace Age, Sex, Profession, Educational Qualification, good Experiences, legal status, number of children, and case history of cervical cancer. The demographics were followed by the questions/statements related to the knowledge on Cervical Cancer, preventive methods of cervical cancer and knowledge on HPV vaccination. For data collection through this survey on a 3 point Likert scale, the respondents recorded their response on the scale of Yes/No/’I don’t know’. The survey had one question aimed at exploring the plausible reasons reported for not administering  HPV and the respondents were instructed to record their choices from pre-determined 6 choices/reasons (Lack of awareness/High cost/Fear of side effects/Doubt on efficacy/Lack of interest/Do not know the importance of the administration of HPV vaccine). The participants could choose more than one option among the choices provided. The reliability of the tool was analysed by using Spearman’s brown prophecy formula (r) = 0.9.

### Sample size computation

The sample size was calculated based on the “nmasters” software. With a power of 80%, α-error of 5% and prevalence rate of 74%, we arrived at the sample size of 264. Adding 20% as nonresponse error, the final sample size was computed to be 317. Statistical significance was considered to be at 5% level.

### Statistical analysis

All the categorical variables are presented as numbers or percentages. Descriptive analysis was performed using univariate statistics to report the Mean and Standard Deviation (SD) for the continuous variable and frequency distributions for the categorical variables. We performed correlation, T-test, and Analysis of Variance (ANOVA) to compare differences in the continuous variables. Pearson chi-square test was used to identify the differences in distribution. The relationship between preventive behaviors (i.e., Pap test or HPV vaccination) against cervical cancer and related factors (such as age, profession, marital status,  number of children,  family history of cervical cancer, and knowledge of cervical cancer) was evaluated using logistic regression analysis. Odds ratios (ORs) and 95% confidence intervals (CIs) were also calculated. All statistical analyses were performed using Statistical Package for Social Science (SPSS, version 17) for Microsoft Windows, SPSS Inc. USA.

## Results

### Socio-demographic characteristics of health care professionals

The socio-demographic characteristics of health care professionals are discussed as follows. Out of the 318 participants (n = 318), 247 were female (77.7%) and 71 were male (22.3%). Around 39.9% of the respondents had less than six months of professional experience. Overall health care professionals who participated in this study were computed to be 53.5%, of which 5.5% were practicing medicine, and 42.2% were nurses. Apart from this, 73% of the participants were observed to be unmarried, and 27% were married professionals (Table 1).

**Table 1:** Demographic characteristics of the health care professional included in the study (N = 318)

Variables	Number	Percent
Age		
< 30 years	254	79.9
≥ 30 years	64	20.1
Sex		
Female	247	77.7
Male	71	22.3
Profession		
Medicine	170	53.5
Dentistry	4	13
Nursing	134	42.2
Allied Heath Science	7	22
Pharmacy	3	9
Education qualification		
Diploma	55	17.3
UG	160	50.3
PG	103	32.4
Professional experience		
Less than 6 months	127	39.9
6 months–2 years	87	27.4
2–6 years	63	19.8
7–10 years	17	5.3
More than 10 years	24	7.5
Marital status		
Married	86	27
Unmarried	232	73
Number of children		
None	27	8.5
One	33	10.4
Two	23	7.2
Three	2	6
Not applicable	233	73.
Family history of cervical cancer		
Yes	6	1.9
No	302	95
Don’t know	8	3.1

### Knowledge of cervical cancer and HPV vaccination

The knowledge survey on cervical cancer among health care professionals revealed that almost 90.6% of the participants were aware of cervical cancer, in which 75.5% of the professionals know that cervical cancer is often prevented by screening. Also, 82.7% of the respondents said 'yes' for cervical cancer leads to mortality and 7.2% of them answered 'don’t know' about the same. Here, 29.2% of the eligible participants were found to have utilized the PAP smear screening against cervical cancer and 24% were vaccinated. Besides this, 94.7% of the study subjects have heard of HPV and 86.2% knew that HPV causes cervical cancer. Among the eligible participants, 83.3% of them knew that PAP smear test is used to detect cervical cancer even before the symptoms appear and 68.2% of the study subjects knew that cancer in the cervix can be prevented by vaccination. Only 34.9% knew that the HPV vaccine could be given to boys. 77.2% of the participants themselves in our study were willing for vaccination and would recommend it to their friends and family members (Tables 2, 3, 4). Among the reasons for not getting the vaccination, 234 participants responded as lack of awareness about HPV vaccination, 164 felt that they did not understand the importance of the vaccination, and 95 participants felt that the vaccine was unaffordable (Tables 3, 4). The Odds ratio and 95% confidence intervals (CI) of family members vaccinated for human papillomavirus vaccination according to selected variables among health care professionals included in the study (N = 318) were recorded as shown in Table 5.

**Table 2:** Knowledge towards cervical cancer among health care professionals enclosed within the study (N = 318)

Variables	Number	Percent
Have you heard about cervical cancer?		
Yes	288	90.6
No	30	9.4
Can screening prevent cervical cancer?		
Yes	240	75.5
No	59	18.6
Don’t know	19	6.0
Is cervical cancer associated with infection?		
Yes	255	80.2
No	43	13.5
Don’t know	20	6.3
Does cervical cancer lead to mortality?		
Yes	263	82.7
No	32	10.1
Don’t know	23	7.2
PAP smear screening is 100% effective		
Yes	197	61.9
No	96	30.2
Don’t know	25	7.9
Is it possible to detect cervical cancer with PAP smear before symptoms appear		
Yes	265	83.3
No	20	6.3
Don’t know	33	10.4
Cervical cancer preventable by vaccination		
Yes	217	68.2
No	71	22.3
Don’t know	30	9.4
Is it possible to cure cervical cancer		
Yes	260	81.8
No	34	10.7
Don’t know	24	7.5
Is early detection of cervical cancer good for treatment outcome		
Yes	297	93.4
No	5	1.6
Don’t know	16	5.0

**Table 3:** Knowledge towards HPV & HPV vaccination among health care professionals enclosed within the study (N = 318)

Variables	Number	Percent
Heard about HPV		
Yes	301	94.7
No	12	3.8
Don’t know	5	1.6
HPV causes cervical cancer		
Yes	274	86.2
No	15	4.7
Don’t know	29	9.1
HPV vaccine can be given to sexually active women		
Yes	180	56.6
No	75	23.6
Don’t know	63	19.8
Can HPV vaccine be given to women who have already having HPV infection		
Yes	90	28.3
No	136	42.8
Don’t know	92	28.9
Can HPV vaccine given to boys		
Yes	111	34.9
No	128	40.3
Don’t know	79	24.8
Can HPV given to pregnant women		
Yes	34	10.7
No	195	61.3
Don’t know	89	28.0
HPV vaccinated women requires screening		
Yes	237	74.5
No	31	9.7
Don’t know	50	15.7
Willingness to receive HPV vaccine and recommendation		
Yes	247	77.7
No	71	22.3
Is HPV vaccine available in India		
Yes	255	80.2
No	11	3.5
Don’t know	52	16.4

**Table 4:** Cervical cancer and HPV vaccination related uptake and barriers of health care professionals (N = 318)

Variables	Number	Percent
Have you or your family members been vaccinated for HPV?		
Yes	63	19.8
No	224	7.4
Don’t know	31	9.7
Reason for not having HPV vaccination		
Lack of awareness	234	73.6
High cost	95	29.9
Fear of side effects	68	21.4
Doubt on efficacy	62	19.5
Lack of interest	70	22.0
Don’t know the importance of administration of HPV vaccine	164	51.6
Ever utilized PAP smear test		
Yes	93	29.2
No	177	55.7
Not applicable	48	15.1

**Table 5:** Odds ratio and 95% confidence intervals of family members vaccinated for human papilomavirus vaccination according to selected variables among health care professionals included in the study (N = 318)

Sl No	Selected variables	Have you or your Family members been vaccinated for HPV? = Yes	Crude OR 95% CI	Adjusted or (95% CI)	Have you or your Family members been vaccinated for HPV? = No	Crude OR 95% CI	Adjusted or (95% CI)
1	Age						
	≤ 30 Yrs 64 (20.1%)	13 (14.1)	1.03	1.78	51 (16.0)	0.99	1.14
	≥ 30 Yrs 254(79.9%)	50 (15.7)			204 (64.2)		
	Odd ratio age (≥ 30 yrs / < 30 yrs)	63 (29.8)	1.040	2.06	–	–	–
2	Profession						
	Paramedical	17 (5.3)	0.44	0.27	.127 (39.9)	1.20	1.08
	Medical	46 (14.5)		0.74	.128(40.3)		1.34
	Odd ratio for Profession (Paramedical/Medical)	0.372	0.20	0.68	–	–	–
3	Professional experience						
	Less than 2 years	44 (13.8)	1.13	0.69	170 (53.5)	0.97	0.87
	More than 2 years	19 (6.0)		1.83	85 (26.7)		1.09
	Odds ratio	1.158	0.64	2.11	–	–	–
4	Marital status						
	Married	16 (5.0)	0.92	0.55	47 (14.8)	1.02	0.91
	Un married	47 (14.8)		1.53	185 (58.2)		1.15
	Odds ratio						
5	Family history of cervical caner						
	Yes	3 (9.)	2.60	1.13	60 (18.9)	0.62	0.278
	No	3 (9)		5.97	252 (79.2)		1.380
	Odds ratio	–	4.20	0.83	–	–	–
6	No. of children						
	One and above	11 (3.5)	0.95	0.53	4.7 (14.8)	1.01	0.88
	None	52 (16.47)		1.70	208 (65.4)		1.16
	Odds ratio	0.936	0.45	1.93	–	–	–
7	Have you heard about cervical cancer						
	Yes	56 (17.6)	0.82	0.42	232 (73.0)	1.05	0.86
	No	7 (2.2)		0.86	23 (7.2)		1.29
	Odds ratio	0.793	0.32	1.94	–	–	–
8	Have you heard of human papiloma virus						
	Yes	61 (19.2)	1.72	0.46	240 (75.)	0.90	0.75
	No	2 (6)		6.46	15 (4.7)		1.09
	Odds ratio	1.906	0.42	8.56	–	–	–
9	PAP smear screening is 100% effective						
	Yes	32 (10..1)	0.63	0.41	165 (51.9)	1.12	0.99
	No	31 (9.7)		0.98	90 (28.3)		1.27
	Odds ratio	0.563	0.32	0.98	–	–	–
10	Did you ever have PAP test						
	Yes	18 (5.7)	0.97	0.59	75 (23.6)		
	No	45 (14.2)		1.58	110 ( 56.6)		
	Odds ratio	0.96	0.52	1.77	–	–	–
11	Cancer in the cervix is preventable by vaccination						
	Yes	49 (15.4)	1.62	94.50	168 (52.8)	0.90	0.80
	No	14(4.4)		2.81	87 (27.4)		1.00
	Odds ratio	1.81	0.95	3.46	–	–	–
12	Can HPV vaccination be given to boys						
	Yes	26 (8.2)	1.31	0.83	85 (26.7)	0.93	0.83
	No	37 (11.6)		2.04	170 (53.5)		1.05
	Odds ratio	1.4	0.79	2.47	–	–	–
13	Lack of awareness						
	Yes	48 (15.1)	1.14	0.68	186 (48.5)	0.97	0.86
	No	15 (4.7)		1.93	69 (21.7)		1.09
	Odds ratio	1.18	0.65	2.25	–	–	–
14	Do not know the importance of the administration of HPV vaccine						
	Yes	33 (10.4)	1.033	0.66	131 (41.2)	0.99	0.88
	No	8.0 (9.4)		1.6	124 (39.0)		1.1
	Odds ratio	1.04	0.6	–	–	–	–

## Discussion

Our study revealed that the majority (90.6%) of the participants were aware of cervical cancer, whereas the awareness index in the studies reported earlier was lesser than that of the participants in this study [9, 10]. When asked about the HPV, 94.7% of the study subjects mentioned that they have heard of it, and 86.2% knew that HPV causes cervical cancer which seems to be better than a similar study in which only 73% of study subjects heard of HPV [9]. A similar study was conducted in Yogyakarta Province in Indonesia from December 2013 to March 2014, which revealed that only 60% of the women participants have heard about cervical cancer [11].

A mixed-method study was conducted in Dhaka, Bangladesh in December 2013 among professional women employed by private banks. It revealed that only 26% of the participants had  heard about cervical cancer and 56.6% of them had  heard about HPV vaccination [12].

In the present study, 83.3% of people knew that PAP smear test detects cervical cancer even before the symptoms appear, which were in comparison with similar studies [13–15]. While 29.2%% of the eligible participants have undergone PAP smear test in our study, it was only 5% in few other studies [16, 17]. This shows that we lack an organized opportunistic screening program for cervical cancer in India. This could be done by making it mandatory to screen all eligible women when they visit health care units for other services. 68.2% of the study subjects knew that cancer cervix could be prevented by vaccination. In our study, 24% of the eligible subjects had the HPV vaccine, which was higher when compared to studies by Swarnapriya et al.[18], Ganju et al. [19], wherein vaccination coverage was 6.8% and 5.5%, respectively. In contrast, 26.73% of them were vaccinated in a study conducted by Hoblidar et al. [20]. We found that awareness regarding the availability of HPV vaccine for boys was also very low (34.9%).

Common reasons for not getting vaccination reported in our study were lack of awareness regarding HPV vaccination, the importance of the vaccine, and high cost which was in agreement with other studies reported earlier [21, 22].

The same finding was also observed in the study conducted by Bhuiyan et al. [12], which also revealed that a lack of knowledge about cervical cancer and HPV has an impact on uptake of HPV vaccination [12]. Also, various studies have reported that people with a family history of genital cancer had shown greater acceptability for vaccination against HPV [22]. 77.2% of the participants in our study were willing for vaccination and recommending the same to their friends and family members, which was in contrast with other studies in which they were not keen on getting vaccinated.1^6^ India is considered to be a slightly conservative country where there are still taboos regarding cervical cancer because HPV infection is predominantly sexually transmitted. Providing information regarding HPV vaccination and screening sensitively can help in the proper execution of these interventions. Unlike other vaccines, the HPV vaccine does not provide 95–100% protection against HPV infection and even after completion of a full course of this high-priced vaccine, one must undergo screening for HPV infection lifelong. All these are seen as drawbacks and reasons for poor uptake.

The profession, knowledge on cervical cancer, HPV screening, and availability of vaccine-associated (statistically significant association) have a negative effect on the administration of HPV vaccination. Knowledge on HPV vaccine to pregnant women associated with showed a positive effect on HPV vaccination. When all the variables were put together, only the variables on the knowledge of HPV vaccination in pregnant women were positively associated with the decision to administer the HPV vaccine or recommend the administration of HPV vaccination to the family and friends.

There is a large gap that has to be filled to improve awareness about HPV vaccination. This could be achieved by promoting awareness emphasizing that every child needs to be vaccinated, and every mother needs to be screened. In developing countries like India, investing and motivating towards preventive measures like vaccination and screening of cervical cancer could help reduce the burden of advanced disease. Since the HPV vaccine is not available to free of cost currently by the Govt of India, its uptake in society is very poor. This needs to be addressed at the earliest because many countries like Australia have nearly eradicated cervical cancer among their population through vaccination and active screening [23]. Conducting surveys and intervention programs to promote vaccination at least annually or opportunistically (during the annual health checkups) could help in developing a positive attitude towards vaccination and screening among health care professionals.

## Limitations

Our study was aimed at identifying the level of awareness among health care professionals in a tertiary care centre catering to the urban population. Hence, these results cannot be generalized to the whole population.

## Conclusion

From our findings in this present study, it is evident that there exists a lack of awareness about HPV vaccination and its importance in preventing cervical cancer among health professionals. This clearly establishes the need to devise intervention programs to promote vaccination against HPV and screening for cervical cancer among healthcare professionals.

## Data Availability

The data used to support the findings of this study are available from the corresponding author upon request.

## Abbreviations

HPV: Human Papilloma Virus
CI: Confidence interval
OR: Odds ratio
PAP: Papanicolaou

## References

[1] Arbyn M, Weiderpass E, Bruni L, de Sanjosé S, Saraiya M, Ferlay J, Bray F (2020). Estimates of incidence and mortality of cervical cancer in 2018: a worldwide analysis. Lancet Glob Health.

[2] Bobdey S, Sathwara J, Jain A, Balasubramaniam G (2016). Burden of cervical cancer and role of screening in India. Indian J Med paediatr oncol.

[3] Kaarthigeyan K (2012). Cervical cancer in India and HPV vaccination. Indian J Med Paediatr Oncol.

[4] Bansal AB, Pakhare AP, Kapoor N, Mehrotra R, Kokane AM (2015). Knowledge, attitude, and practices related to cervical cancer among adult women: A hospital-based cross-sectional study. J Nat Sci Biol Med.

[5] Srivastava AN, Misra JS, Srivastava S, Das BC, Gupta S (2018). Cervical cancer screening in rural India: Status & current concepts. Indian J Med Res.

[6] Schiller JT, Lowy DR (2000). Papillomavirus-like particle vaccines. J Natl Cancer Inst Monographs.

[7] Markowitz LE, Dunne EF, Saraiya M, Lawson HW, Chesson H, Unger ER (2007). Quadrivalent Human Papillomavirus Vaccine: Recommendations of the Advisory Committee on Immunization Practices (ACIP). MMWR Recomm Rep.

[8] Singhal T (2008). Indian Academy of Pediatrics Committee on Immunisation (IAPCOI)—Consensus Recommendations on Immunization 2008. Indian Pediatr.

[9] Ali SF, Ayub S, Manzoor NF, Azim S, Afif M, Akhtar N, Jafery WA, Tahir I, Farid-ul-Hasnian S, Uddin N (2010). Knowledge and awareness about cervical cancer and its prevention amongst interns and nursing staff in Tertiary Care Hospitals in Karachi, Pakistan. PLoS ONE.

[10] Tandon A, Raja S, Pai MM, Unnikrishnan B, Kanchan T (2019). Knowledge and awareness of the cause, prevention and control of cervical cancer amongst female undergraduates and faculty of health sciences: a cross sectional survey. Int J Reprod Contracept Obstet Gynecol.

[11] Endarti D, Satibi S, Kristina SA, Farida MA, Rahmawanti Y, Andriani T (2018). Knowledge, perception, and acceptance of HPV vaccination and screening for cervical cancer among women in Yogyakarta Province. Indonesia Asian Pac J Cancer Prev.

[12] Bhuiyan A, Sultana F, Islam JY, Chowdhury MAK, Nahar Q (2018). Knowledge and acceptance of human papillomavirus vaccine for cervical cancer prevention among urban professional women in Bangladesh: a mixed-method study. Biores Open Access.

[13] Singh E, Seth S, Rani V, Srivastava DK (2012). Awareness of cervical cancer screening among nursing staff in a tertiary institution of rural India. J Gynecol Oncol.

[14] Mutyaba T, Mmiro FA, Weiderpass E (2006). Knowledge, attitudes and practices on cervical cancer screening among the medical workers of Mulago Hospital, Uganda. BMC Med Educ.

[15] Anya SE, Oshi DC, Nwosu SO, Anya AE (2005). Knowledge, attitude, and practice of female health professionals regarding cervical cancer and Pap smear. Niger J Med.

[16] Udigwe GO (2006). Knowledge, attitude and practice of cervical cancer screening (pap smear) among female nurses in Nnewi, South Eastern Nigeria. Niger J Clin Pract.

[17] Bandekar PK, Kale PB (2018). Awarness of Carcinoma cervix in nursing personnel of a tertiary care institute, Mumbai, India. Int J Reprod Contracept Obstet Gynecol.

[18] Swarnapriya K, Kavitha D, Reddy GM (2015). Knowledge, attitude and practices regarding HPV vaccination among medical and para medical in students, India a cross sectional study. Asian Pac J Cancer Prev.

[19] Ganju SA, Gautam N, Barwal V, Walia S, Ganju S (2017). Assessment of knowledge and attitude of medical and nursing students towards screening for cervical carcinoma and HPV vaccination in a tertiary care teaching hospital. Int J Community Med Public Health.

[20] Hoblidar S, Moni SS, Desai RM, Nera A (2020). Human papilloma virus vaccination: knowledge, awareness and acceptability among medical and paramedical students. Int J Reprod Contracept Obstet Gynecol.

[21] Das EN, Francis PT (2018). HPV vaccine knowledge and coverage among female students in a medical college, Kerala. Int J Community Med Public Heal.

[22] Di Giuseppe G, Abbate R, Liguori G, Albano L, Angelillo IF (2008). Human papillomavirus and vaccination: knowledge, attitudes, and behavioural intention in adolescents and young women in Italy. Br J Cancer.

[23] Bralsford KJ, Jamieson E (2019). Following Australia’s lead to eradicate cervical cancer. BMJ.

